# Uninfected Bystander Cells Impact the Measurement of HIV-Specific Antibody-Dependent Cellular Cytotoxicity Responses

**DOI:** 10.1128/mBio.00358-18

**Published:** 2018-03-20

**Authors:** Jonathan Richard, Jérémie Prévost, Amy E. Baxter, Benjamin von Bredow, Shilei Ding, Halima Medjahed, Gloria G. Delgado, Nathalie Brassard, Christina M. Stürzel, Frank Kirchhoff, Beatrice H. Hahn, Matthew S. Parsons, Daniel E. Kaufmann, David T. Evans, Andrés Finzi

**Affiliations:** a Centre de Recherche du CHUM, Montreal, Quebec, Canada; b Department of Microbiology, Infectiology and Immunology, Université de Montréal, Montreal, Quebec, Canada; c Department of Pathology and Laboratory Medicine, University of Wisconsin, Madison, Wisconsin, USA; d Institute of Molecular Virology, Ulm University Medical Center, Ulm, Germany; e Departments of Medicine and Microbiology, Perelman School of Medicine, University of Pennsylvania, Philadelphia, Pennsylvania, USA; f Department of Microbiology and Immunology, The University of Melbourne, at The Peter Doherty Institute for Infection and Immunity, Melbourne, Victoria, Australia; g Department of Medicine, Université de Montréal, Montreal, Quebec, Canada; h Center for HIV/AIDS Vaccine Immunology and Immunogen Discovery, The Scripps Research Institute, La Jolla, California, USA; i Wisconsin National Primate Research Center, University of Wisconsin, Madison, Wisconsin, USA; j Department of Microbiology and Immunology, McGill University, Montreal, Quebec, Canada; Columbia University

**Keywords:** A32, ADCC, ADCC assay, CD4i Abs, Env, granzyme B assay, HIV-1, luciferase assay, RFADCC, uninfected bystander, bNAbs

## Abstract

The conformation of the HIV-1 envelope glycoprotein (Env) substantially impacts antibody recognition and antibody-dependent cellular cytotoxicity (ADCC) responses. In the absence of the CD4 receptor at the cell surface, primary Envs sample a “closed” conformation that occludes CD4-induced (CD4i) epitopes. The virus controls CD4 expression through the actions of Nef and Vpu accessory proteins, thus protecting infected cells from ADCC responses. However, gp120 shed from infected cells can bind to CD4 present on uninfected bystander cells, sensitizing them to ADCC mediated by CD4i antibodies (Abs). Therefore, we hypothesized that these bystander cells could impact the interpretation of ADCC measurements. To investigate this, we evaluated the ability of antibodies to CD4i epitopes and broadly neutralizing Abs (bNAbs) to mediate ADCC measured by five ADCC assays commonly used in the field. Our results indicate that the uninfected bystander cells coated with gp120 are efficiently recognized by the CD4i ligands but not the bNabs. Consequently, the uninfected bystander cells substantially affect *in vitro* measurements made with ADCC assays that fail to identify responses against infected versus uninfected cells. Moreover, using an mRNA flow technique that detects productively infected cells, we found that the vast majority of HIV-1-infected cells in *in vitro* cultures or *ex vivo* samples from HIV-1-infected individuals are CD4 negative and therefore do not expose significant levels of CD4i epitopes. Altogether, our results indicate that ADCC assays unable to differentiate responses against infected versus uninfected cells overestimate responses mediated by CD4i ligands.

## IMPORTANCE

Emerging evidence supports a role for antibody-dependent cellular cytotoxicity (ADCC) in protection against HIV-1 transmission and disease progression. However, there are conflicting reports regarding the ability of nonneutralizing antibodies targeting CD4-inducible (CD4i) Env epitopes to mediate ADCC. Here, we performed a side-by-side comparison of different methods currently being used in the field to measure ADCC responses to HIV-1. We found that assays which are unable to differentiate virus-infected from uninfected cells greatly overestimate ADCC responses mediated by antibodies to CD4i epitopes and underestimate responses mediated by broadly neutralizing antibodies (bNAbs). Our results strongly argue for the use of assays that measure ADCC against HIV-1-infected cells expressing physiologically relevant conformations of Env to evaluate correlates of protection in vaccine trials.

## INTRODUCTION

Antibody-dependent cellular cytotoxicity (ADCC) represents a major effector mechanism used by the immune system to target and eliminate virally infected cells. Besides being incorporated into viral particles, the HIV-1 envelope glycoprotein (Env) trimer represents the only virus-specific target exposed on the surface of infected cells and thus represents a major target for ADCC (1). Emerging evidence suggests that Env conformation plays a critical role in the susceptibility of HIV-1-infected cells to ADCC (2, 3). HIV-1 Env is a metastable molecule, which is driven by CD4 receptor engagement to transition from its unliganded “closed” high-energy conformation (state 1) into an intermediate “partially open” conformation (state 2) and then into a more open CD4-bound conformation (state 3) (4). Interaction of Env with the CD4 receptor was reported to be critical for the exposure of epitopes for ADCC-mediating antibodies (Abs) (5–7). Accordingly, ADCC-mediating Abs naturally present in sera from HIV-1-infected individuals (HIV^+^ sera) preferentially target HIV-1-infected cells that present Env in states 2 and 3 (5, 8). In line with this observation, ADCC activity present in sera from HIV-1-infected individuals (HIV^+^ sera) is predominantly mediated by the anti-cluster A Abs (5, 9–11, 14). These nonneutralizing antibodies (nnAbs) target a highly conserved region in the gp120 inner domain that is buried inside the closed unliganded Env and becomes exposed only upon CD4 engagement (6, 7, 10–14). Thus, cells infected with primary viruses that expose Env in its closed unliganded conformation are largely resistant to ADCC induced by these nnAbs (7, 10, 15–19).

To protect HIV-1-infected cells from ADCC by naturally occurring CD4-induced (CD4i) Abs, the virus has evolved several strategies to limit the adoption of the CD4-bound conformation and thus prevent exposure of vulnerable CD4i epitopes. HIV-1 limits Env-CD4 interaction by both downregulating CD4 and preventing Env accumulation at the surface of infected cells (5, 7, 20–22). Two accessory proteins, Nef and Vpu, reduce cell surface expression of CD4 (5, 7), while Env accumulation is tightly controlled through efficient internalization (22) and Vpu-mediated BST-2 downregulation (20, 21, 23). Therefore, Nef and Vpu play a central role in protecting HIV-infected cells from ADCC by averting the premature exposure of vulnerable epitopes.

While HIV-1-infected cells are generally protected from ADCC, we recently found that uninfected bystander CD4^+^ T cells are susceptible to ADCC mediated by CD4i ligands (16). It has been well established that due to its noncovalent association with gp41, gp120 sheds from the surface of productively infected cells (13, 24, 25). Binding of shed gp120 to the CD4 receptor on the surface of uninfected bystander cells exposes vulnerable CD4i ADCC epitopes and results in the sensitization of these cells to ADCC (16). However, the extent to which exposure of these CD4i epitopes on uninfected bystander cells impacts *in vitro* measurements of ADCC has not yet been determined. Many ADCC assays measure killing of total cell population and thus are unable to differentiate ADCC responses against HIV-infected cells from those against uninfected bystander cells. Here, we compared different ADCC assays currently used in the field for their ability to measure HIV-1-infected cell-specific responses. We found that uninfected bystander cells greatly impact *in vitro* measurements of ADCC by introducing a significant bias toward CD4i Abs.

## RESULTS

### Differential recognition of uninfected bystander cells and infected cells by ADCC-mediating Abs.

**FIG 1:**
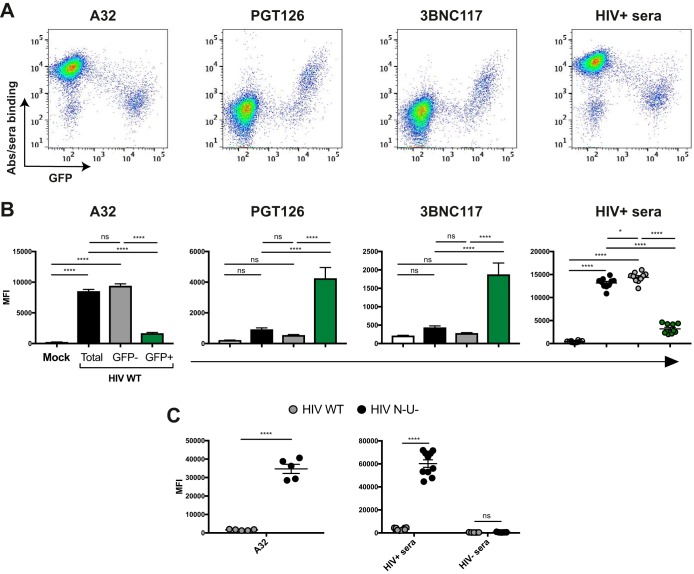
Differential recognition of infected and uninfected bystander cells by ADCC-mediating Abs. Primary CD4^+^ T cells were mock infected or infected with the NL4.3 ADA GFP virus, either wild type (HIV WT) or defective for Nef and Vpu expression (HIV N^−^ U^−^). Forty-eight hours postinfection, cells were stained with the anti-Env Ab (5 μg/ml) A32, PGT126, or 3BNC117 or sera (1:1,000 dilution) from 10 HIV-1-infected (HIV^+^ sera) or 5 uninfected (HIV^−^ sera) individuals, followed by appropriate secondary Abs. (A) Dot plots depicting representative staining of WT-infected cells. (B) Mean fluorescence intensities (MFI) obtained for at least 5 independent stainings with the different Abs and 10 HIV^+^ or 5 HIV^−^ sera. (C) Graphs represent the MFI obtained for 5 independent staining experiments with A32 and 10 HIV^+^ or 5 HIV^−^ sera on cells infected with WT and N^−^ U^−^ virus. Error bars indicate means ± standard errors of the means. Statistical significance was tested using ordinary one-way analysis of variance (B) or unpaired *t* test or Mann-Whitney test (C) (*, *P* < 0.05; ****, *P* < 0.0001; ns, nonsignificant).

We first explored the capacity of different ADCC-mediating Abs to recognize uninfected bystander cells versus productively infected cells. To this end, we infected primary CD4^+^ T cells from HIV-1-uninfected individuals with a previously reported wild-type (WT) HIV-1 strain that encodes all accessory proteins as well as a *gfp* reporter gene and the R5-tropic (ADA) envelope (NL4.3 ADA green fluorescent protein [GFP]) (7, 16). In this system, productively infected cells are GFP^+^, whereas GFP^−^ cells represent the uninfected bystander cells. Forty-eight hours postinfection, the average percentage of infected cells was 12.6%. At this step, cells were incubated with HIV^+^ sera, the nnAb A32, or a broadly neutralizing Ab (bNAb) (either PGT126 or 3BNC117). The cluster A-specific monoclonal antibody (MAb) A32 recognizes a highly conserved CD4i epitope located at the interface of the gp120 inner domain layers 1 and 2 (7, 11–13). As previously reported, productively infected (GFP^+^) cells were poorly recognized by A32 as well as HIV^+^ sera (16), while mock-infected cells were not recognized (Fig. 1A to C). This weak recognition of infected cells is likely due to the efficient downregulation of CD4 by Nef and Vpu (see Fig. S1 in the supplemental material), which permits Env to retain its “closed” conformation. In contrast, uninfected bystander (GFP^−^) cells from the same culture were readily recognized by A32 and HIV^+^ sera (Fig. 1A and B). As most cells present in the culture are gp120-coated uninfected bystander cells (16), strong binding was detected for A32 and HIV^+^ sera when Ab binding was measured for the total cell population (i.e., both uninfected and infected cells) (Fig. 1B). Of note, sera from HIV-1-uninfected individuals (HIV^−^ sera) did not react with any cell population (Fig. 1C).

10.1128/mBio.00358-18.2 FIG S1  Level of cell surface CD4 on infected and uninfected cells. Download FIG S1, PDF file, 0.7 MB. Copyright © 2018 Richard et al. 2018 Richard et al. This content is distributed under the terms of the Creative Commons Attribution 4.0 International license.

In contrast to nnAbs, bNAbs preferentially recognize Env in its closed conformation (4). PGT126 binds a conserved region at the V3 loop stem near the N332 glycosylation site (26–28), while 3BNC117 recognizes the CD4-binding site (29). Both bNAbs were previously found to mediate ADCC against HIV-1-infected cells (18, 21, 30–32). Consistent with these findings, PGT126 and 3BNC117 efficiently recognized productively infected cells (GFP^+^) but not the uninfected GFP^−^ cells (Fig. 1A and B). As expected, since the majority of the cells in the culture are not recognized by these Abs, the overall (total) signal obtained with these Abs was lower than the signal obtained with A32 or HIV^+^ sera (Fig. 1B). In agreement with the role of Nef and Vpu in preventing the formation of CD4i epitopes through CD4 downregulation (Fig. S1), deletion of these accessory genes dramatically increased recognition of infected (GFP^+^) cells by A32 and HIV^+^ sera (Fig. 1C). To rule out the possibility that these phenotypes were related to the viral strain used, we also used primary CD4^+^ T cells infected with the transmitted founder (TF) virus CH77 and obtained similar recognition patterns (Fig. S2). Altogether, these results indicate that CD4i ligands recognize uninfected bystander cells coated with shed gp120 more efficiently than Abs preferentially recognizing the closed trimer.

10.1128/mBio.00358-18.3 FIG S2  Recognition of primary CD4^+^ T cells infected with the transmitted founder virus CH77. Download FIG S2, PDF file, 0.7 MB. Copyright © 2018 Richard et al. 2018 Richard et al. This content is distributed under the terms of the Creative Commons Attribution 4.0 International license.

### Assays measuring ADCC against productively infected cells reveal greater killing of infected cells by bNAbs than by CD4i Abs.

**FIG 2:**
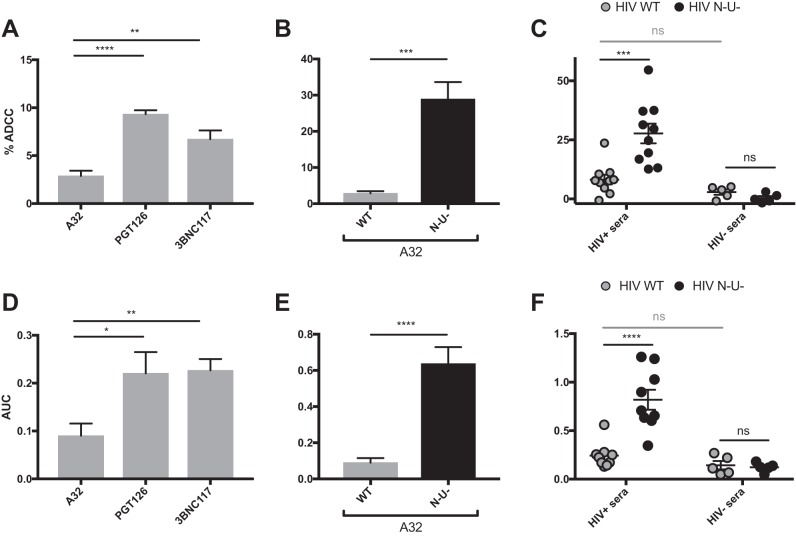
ADCC responses detected with assays measuring the elimination of infected cells. Primary CD4^+^ T cells (A to C) or CEM.NKr-CCR5-sLTR-Luc cells (D to F) infected with the NL4.3 ADA GFP virus, either wild-type (HIV WT) (depicted in gray) or defective for Nef and Vpu expression (HIV N^−^ U^−^) (depicted in black) were used as target cells with the FACS-based infected-cell elimination assay (A to C) or the luciferase assays (D to F). (A and D) ADCC responses detected with the anti-Env Abs A32, PGT126, and 3BNC117 against cells infected with the WT virus. (B, C, E, and F) ADCC responses detected with A32 (B and E) or HIV^+^ and HIV^−^ sera (C and F) against cells infected with WT or N^−^ U^−^ viruses. All graphs shown represent ADCC responses obtained from at least 5 independent experiments. For the FACS-based assay, MAbs were used at 5 μg/ml and human sera were used at a 1:1,000 dilution. For the luciferase assay, area under the curve (AUC) values were calculated using increased concentrations of MAbs (0.0024, 0.0098, 0.0390, 0.1563, 0.6250, 2.5, and 10 μg/ml) and increased dilutions of human sera (1:100, 1:400, 1:1,600, 1:6,400, 1:25,600, and 1:102,400). Error bars indicate means ± standard errors of the means. Statistical significance was tested using unpaired *t* test or Mann-Whitney test (*, *P* < 0.05; **, *P* < 0.01; ***, *P* < 0.001; ****, *P* < 0.0001; ns, nonsignificant).

To evaluate the potential impact of the uninfected bystander cell population on ADCC, we compared different assays currently used in the field to detect ADCC responses against WT-infected cells using the A32, PGT126, or 3BNC117 MAb or human sera. We initially tested assays designed to distinguish ADCC responses against infected cells from those against uninfected bystander cells. These included the fluorescence-activated cell sorting (FACS)-based infected-cell elimination (ICE) assay, in which ADCC-mediated elimination of productively infected cells is determined by calculating the loss of infected cells using a GFP-expressing virus (5, 7, 10, 16) or by measuring intracellular HIV-1 p24 antigen (10, 15, 17, 32). Using primary CD4^+^ T cells infected with the NL4.3 ADA GFP WT virus as target cells and autologous peripheral blood mononuclear cells (PBMCs) as effector cells, we found that WT-infected cells were significantly more susceptible to ADCC mediated by PGT126 and 3BNC117 than to that mediated by A32 (Fig. 2A). Furthermore, WT-infected cells were largely resistant to ADCC responses mediated by A32 (Fig. 2B, gray bars) and responses mediated by HIV^+^ sera were comparable to those seen with HIV^−^ sera (Fig. 2C, gray circles). Again, deletion of *nef* and *vpu* genes drastically increased ADCC responses mediated by A32 and HIV^+^ sera (Fig. 2B and C, black bars and circles, respectively), confirming the dependence of this killing on Env-CD4 interaction.

Measurement of ADCC-mediated elimination of infected cells was also conducted using a luciferase assay (33). In this assay, infected CEM.NKr-CCR5-sLTR-Luc cells expressing a Tat-driven luciferase reporter gene serve as target cells, while human PBMCs or a CD16^+^ NK cell line is used as effector cells (18, 21, 22, 33). As luciferase is expressed only upon productive HIV-1 infection, elimination of infected cells can be calculated by the loss of luciferase activity. Since this assay measures the elimination of productively infected (Tat-expressing) cells, we observed ADCC responses very similar to those obtained with the FACS-based ICE assay (compare Fig. 2A to C with D to F; Fig. S3). ADCC responses mediated by PGT126 and 3BNC117 were significantly higher than those obtained with A32. HIV^+^ sera and A32 mediated robust ADCC responses only against cells infected with the *nef*^−^ *vpu*^−^ virus (Fig. 2E and F). Similar results were obtained using target cells infected with the transmitted founder CH77 virus (Fig. S4). These results confirm the increased ability of bNAbs to mediate ADCC responses against infected cells compared to CD4i Abs.

10.1128/mBio.00358-18.4 FIG S3  ADCC responses detected by the luciferase assays. Download FIG S3, PDF file, 0.7 MB. Copyright © 2018 Richard et al. 2018 Richard et al. This content is distributed under the terms of the Creative Commons Attribution 4.0 International license.

10.1128/mBio.00358-18.5 FIG S4  ADCC responses detected with the FACS-based and luciferase assays against cells infected with the transmitted founder virus CH77. Download FIG S4, PDF file, 0.7 MB. Copyright © 2018 Richard et al. 2018 Richard et al. This content is distributed under the terms of the Creative Commons Attribution 4.0 International license.

10.1128/mBio.00358-18.6 FIG S5  Gating strategy used for the NK cell activation and granzyme B assays. Download FIG S5, PDF file, 1 MB. Copyright © 2018 Richard et al. 2018 Richard et al. This content is distributed under the terms of the Creative Commons Attribution 4.0 International license.

10.1128/mBio.00358-18.7 FIG S6  Gating strategy used for the RFADCC assays. Download FIG S6, PDF file, 0.8 MB. Copyright © 2018 Richard et al. 2018 Richard et al. This content is distributed under the terms of the Creative Commons Attribution 4.0 International license.

### Assays measuring ADCC activities on the total cell population overestimate the responses mediated by CD4i Abs.

The two assays described above are able to distinguish between HIV-1-infected and uninfected bystander cells. Other ADCC methods, however, assess killing on the total cell population (i.e., uninfected and infected cells). Given that the binding of shed gp120 on uninfected bystander cells enables recognition of these cells by CD4i Abs but not bNAbs, we hypothesized that these assays would primarily detect killing of bystander cells.

**FIG 3:**
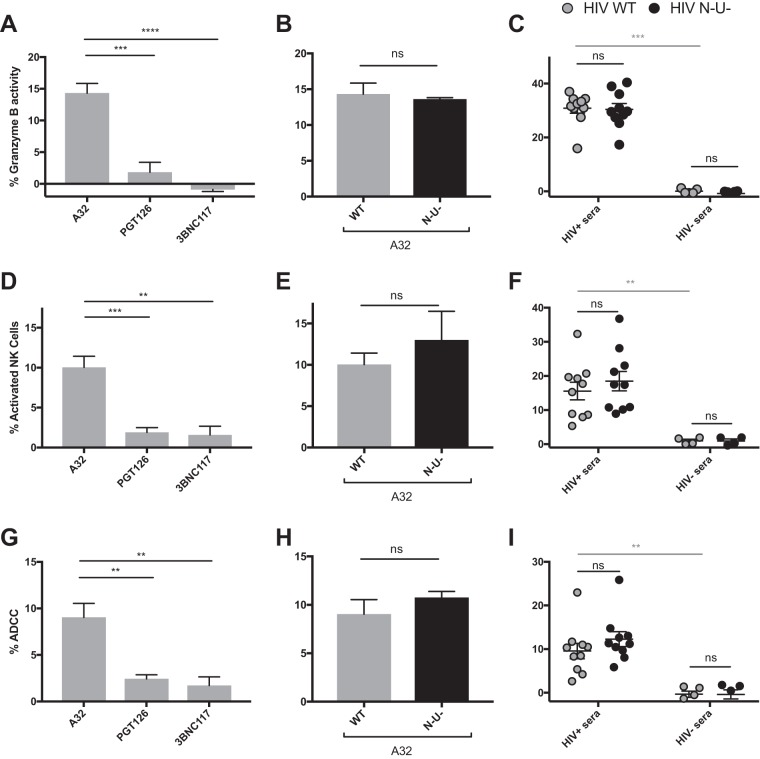
ADCC responses detected with assays relying on the total cell population. Primary CD4^+^ T cells infected with the NL4.3 ADA GFP virus, either wild-type (HIV WT) (depicted in gray) or defective for Nef and Vpu expression (HIV N^−^ U^−^) (depicted in black), were used as target cells in the granzyme B assay (A to C), the NK cell activation assay (D to F), or FACS-based assays (gating on the total cell population) (G to I). (A, D, and G) ADCC responses detected with the anti-Env MAbs (5 μg/ml) A32, PGT126, and 3BNC117 against cells infected with WT virus. (B, C, E, F, H, and I) ADCC responses mediated by A32 (B, E, and H) or HIV^+^ and HIV^−^ sera (1:1,000 dilution) (C, F, and I) against cells infected with WT or N^−^ U^−^ virus. All graphs shown represent ADCC responses obtained for at least 5 independent experiments. Error bars indicate means ± standard errors of the means. Statistical significance was tested using unpaired *t* test or Mann-Whitney test (*, *P* < 0.05; **, *P* < 0.01; ***, *P* < 0.001; ****, *P* < 0.0001; ns, nonsignificant).

**FIG 4:**
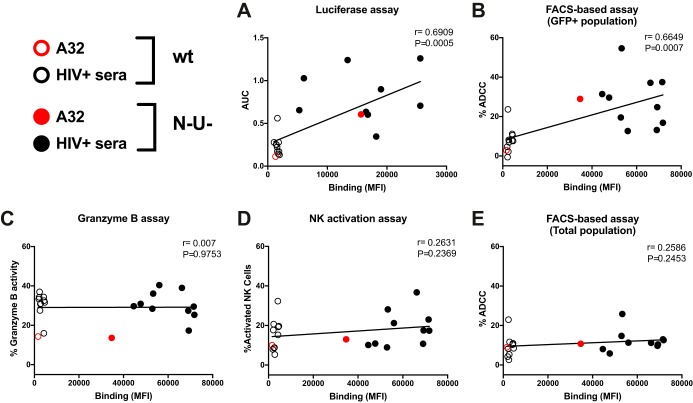
Recognition of infected cells correlates with ADCC responses when using assays measuring the elimination of the infected-cell population. Correlation between the ability of A32 and HIV^+^ sera to recognize cells infected with NL4.3 ADA GFP, either wild-type (WT) or defective for Nef and Vpu expression (N^−^ U^−^), and the ADCC responses detected against these cells using the luciferase assays (A), the FACS-based assay (on the GFP^+^ cell population) (B), the granzyme B assay (C), the NK cell activation assay (D), or the FACS-based assay (E) (on the total cell population) was calculated using a Pearson correlation test.

To investigate this, we performed a similar series of experiments as in Fig. 2 but measured ADCC using assays that detect killing within the total cell population: the granzyme B assay and the NK cell activation assay. The granzyme B assay (GranToxiLux or Pantoxilux assay) detects granzyme B activity in target cells upon incubation with NK cells and Abs or sera (34–36). Since this assay is not compatible with the permeabilization step required to perform intracellular p24 staining, the user cannot differentiate productively infected cells from uninfected bystander cells. Similarly, the NK cell activation assay, which measures NK activation markers (CD107a and interferon gamma [IFN-γ]), is unable to determine which cell population (infected or uninfected) leads to NK cell activation (20, 37–40). The ADCC responses detected with the granzyme B and NK cell activation assays were strikingly different from those measured with the FACS-based ICE and luciferase assays (compare Fig. 2 to 3A to F). Strong responses were detected against WT-infected targets using A32, while weak responses were observed with PGT126 and 3BNC117 (Fig. 3A and D). Similarly, robust granzyme B activity and NK cell activation were detected with HIV^+^ sera in the context of WT-infected target cells but not with HIV^−^ sera (Fig. 3C and F), while both assays were unable to detect the protective effect of Nef and Vpu accessory proteins on ADCC responses (Fig. 3B, C, E, and F and S5). Results obtained with granzyme B and NK activation were similar to those obtained with the FACS-based ICE assay when responses were calculated for the total population (GFP^−^ and GFP^+^) rather than by gating on productively infected (GFP^+^) cells (Fig. 3G to I). Moreover, while assays measuring the elimination of productively infected cells (FACS-based and luciferase assays) showed a positive correlation between antibody binding and ADCC (Fig. 4A and B), no such correlation was observed with assays that measured killing of total targets (granzyme B, NK activation, or FACS-based ICE on total cell population) (Fig. 4C to E). Thus, assays relying on the assessment of ADCC responses on the total cell population overestimate ADCC responses mediated by CD4i Abs and at the same time underestimate responses mediated by bNAbs.

### Most ADCC activity detected using total cell target population is directed against uninfected bystander cells.

**FIG 5:**
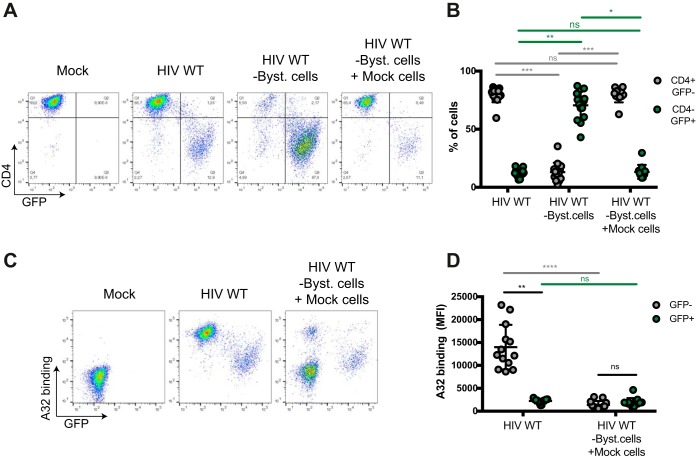
Replacement of uninfected bystander cells by autologous mock-infected cells reduces the proportion of cells recognized by A32. Primary CD4^+^ T cells were mock infected (Mock) or infected with the NL4.3 ADA GFP WT virus (HIV WT). Forty-eight hours postinfection, uninfected bystander CD4^+^ cells were removed (HIV WT -Byst. cells) and replaced by the same number of autologous mock-infected cells (HIV WT -Byst. cells + Mock cells). Cells were stained with anti-CD4 (1 μg/ml) and A32 (5 μg/ml) Abs. (A and C) Representative staining for CD4 (A) and A32 (C). (B) Percentage of CD4^+^ GFP^−^ and CD4^−^ GFP^+^ cells. (D) MFI obtained for the A32 staining for at least 13 independent experiments. Error bars indicate means ± standard errors of the means. Statistical significance was tested using a Kruskal-Wallis test (*, *P* < 0.05; **, *P* < 0.01; ***, *P* < 0.001; ****, *P* < 0.0001; ns, nonsignificant).

**FIG 6:**
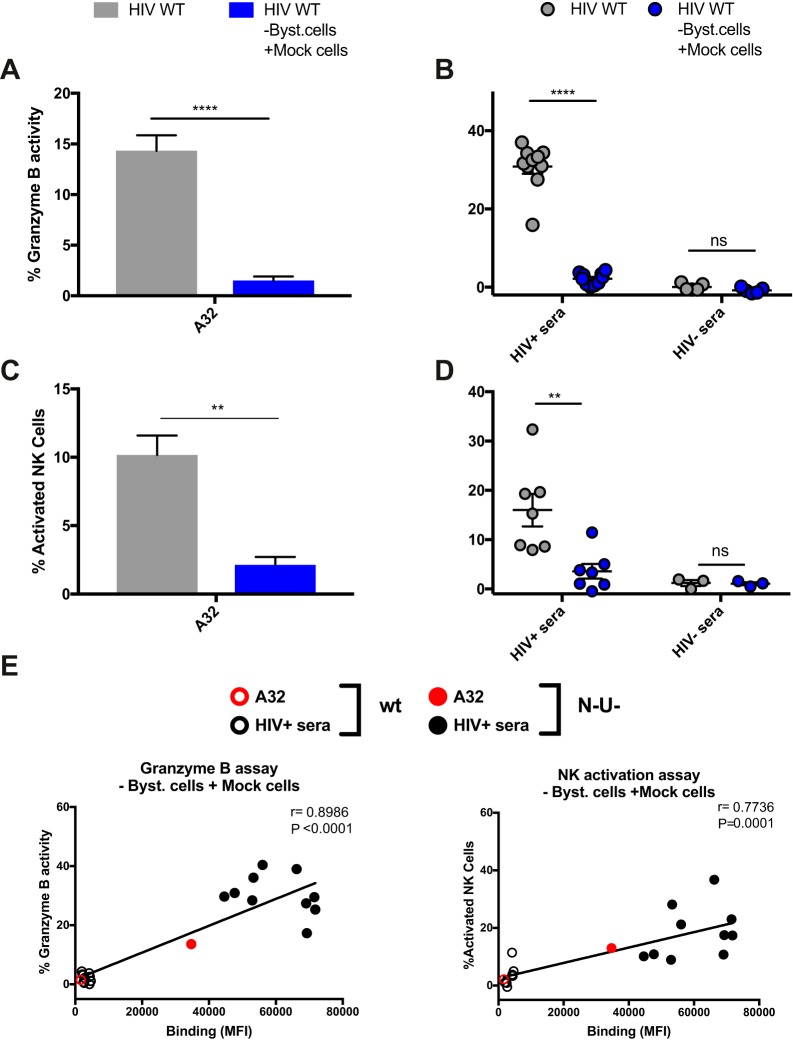
Replacement of uninfected bystander cells by autologous mock-infected cells strongly reduces the ADCC responses detected with granzyme B and NK cell activation assays. Primary CD4^+^ T cells were mock infected (Mock) or infected with the NL4.3 ADA GFP WT virus (HIV WT). Forty-eight hours postinfection, uninfected bystander CD4^+^ T cells were removed and replaced by the same number of autologous mock-infected cells (HIV WT -Byst. cells +Mock cells) prior to ADCC measurements with the granzyme B assay (A and B) and the NK cell activation assay (C and D). (A and C) ADCC responses detected with A32 (5 μg/ml). (B and D) Responses mediated by HIV^+^ and HIV^−^ sera (1:1,000 dilution). (E) A correlation between the ability of A32 and HIV^+^ sera to recognize infected cells and the ADCC responses detected with the granzyme B and NK cell activation assay was observed when the uninfected bystander CD4^+^ T cells were replaced by autologous mock-infected cells in the context of a WT infection. All graphs shown represent ADCC responses obtained in at least 5 independent experiments. Error bars indicate means ± standard errors of the means. Statistical significance was tested using unpaired *t* test or Mann-Whitney test (A to D) and a Pearson correlation test (E) (**, *P* < 0.01; ****, *P* < 0.0001; ns, nonsignificant).

Since A32 and HIV^+^ sera preferentially recognize uninfected bystander cells (Fig. 1), we hypothesized that most of the ADCC responses detected with the granzyme B and NK activation assays were directed against such cells. To test this possibility, uninfected bystander cells (GFP^−^ CD4^+^) were removed from the infected coculture using beads coated with an anti-CD4 antibody that does not compete for gp120 binding (see Materials and Methods) (Fig. 5A). These uninfected bystander cells were replaced by the same number of autologous mock-infected cells (i.e., never exposed to HIV) prior to ADCC measurements. Importantly, this procedure did not affect the percentage of productively infected cells (percent GFP^+^ CD4^−^) in the cell culture (Fig. 5A and B). As expected, the replacement of uninfected bystander cells by mock-infected cells did not alter recognition of infected GFP^+^ cells but decreased the proportion of uninfected bystander cells recognized by A32 (Fig. 5C and D). This replacement also dramatically reduced ADCC responses mediated by both A32 and HIV^+^ sera using both the granzyme B and NK cell activation assays (Fig. 6). Finally, removal of bystander cells resulted in a positive correlation between the abilities of A32 and HIV^+^ sera to recognize infected cells and trigger ADCC responses (Fig. 6E). Thus, uninfected bystander cells greatly influence the measurement of ADCC responses by assays that cannot distinguish infected from uninfected cells.

### Measurement of ADCC responses using gp120-coated cells preferentially detects CD4i-mediated ADCC responses.

**FIG 7:**
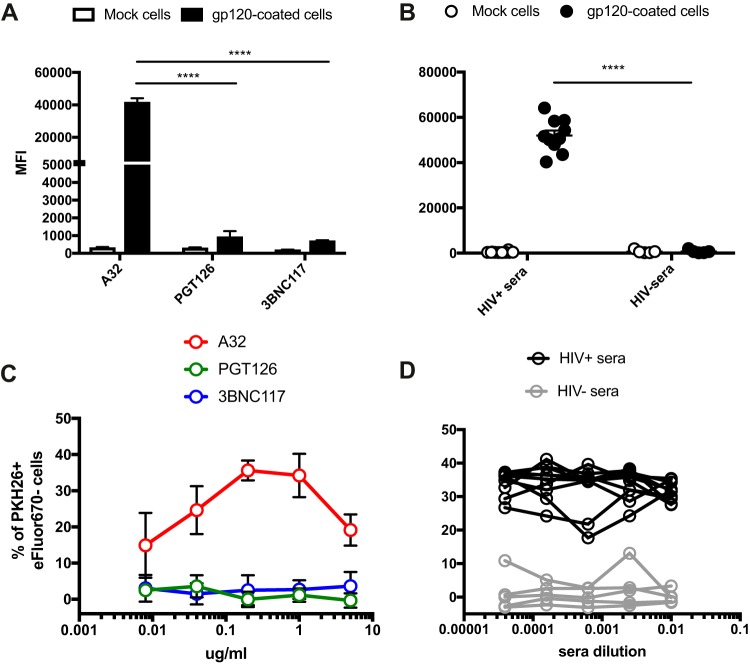
Measurement of ADCC responses against gp120-coated target cells. (A and B) Recognition of gp120-coated CEM.NKr cells by A32, PGT126, or 3BNC117 (A) and HIV^+^ or HIV^−^ sera (B). (C and D) ADCC responses detected using the RFADCC assay against gp120-coated cells with the anti-Env Ab A32, PGT126, or 3BNC117 (0.008, 0.04, 0.2, 1, and 5 μg/ml) (C) and HIV^+^ or HIV^−^ sera (1:100, 1:400, 1:1,600, 1:6,400, and 1:25,600 dilutions) (D). All graphs shown represent staining and ADCC responses obtained in at least 3 independent experiments. Error bars indicate means ± standard errors of the means. Statistical significance was tested using unpaired *t* test (****, *P* < 0.0001).

Cells coated with recombinant gp120 are frequently used as target cells to assess the ADCC activity of monoclonal antibodies (MAbs) or sera from HIV-1-infected or vaccinated individuals (9, 14, 34, 40–50). In these assays, CD4^+^ target cells are incubated with recombinant gp120 monomers, which adopt a CD4-bound conformation on the target cells and expose surfaces of the protein that are normally occluded in native Env trimers (51). We thus evaluated Ab binding and ADCC responses using gp120-coated target cells. The NK cell-resistant cell line CEM.NKr was coated with recombinant gp120 and subsequently used as target cells to measure ADCC (41). As predicted from results in Fig. 1, gp120-coated CEM.NKr cells were efficiently recognized by A32 and HIV^+^ sera but not by HIV^−^ sera (Fig. 7A and B) or PGT126 and 3BNC117 (Fig. 7A and B). This was also the case when the rapid fluorometric ADCC assay (RFADCC assay) (41), which uses gp120-coated target cells to detect ADCC responses, was used for analysis (12, 14, 40, 43, 44, 52). As presented in Fig. 7C and D and S6, robust responses were detected with A32 and HIV^+^ sera but not with PGT126, 3BNC117, or HIV^−^ sera (Fig. 7C and D). Thus, gp120-coated target cells detected ADCC responses largely mediated by CD4i antibodies and not by bNAbs capable of recognizing functional Env trimers, such as antibodies to the CD4-binding site or to a proteoglycan epitope in the V3 region.

### A32 preferentially recognizes CD4^+^ p24^−^ cells not expressing HIV-1 *gag-pol* mRNA.

Our results suggest that A32 preferentially targets uninfected bystander cells rather than productively infected cells (Fig. 2 and 5), although anti-cluster A Abs, such as A32, were initially identified as potent ADCC-mediating Abs (9, 14). Therefore, we could not exclude the possibility that the cells detected as bystander cells in our assays were infected but below the limit of detection. To investigate this possibility, we used a previously described RNA-flow fluorescence in situ hybridization (FISH) method (53, 54). This method identifies productively infected cells by visualizing cellular HIV-1 *gag-pol* mRNA by *in situ* RNA hybridization and intracellular Ab staining for the HIV-1 p24 protein. This approach is 1,000-fold more sensitive than p24 staining alone, with a detection limit of 0.5 to 1 *gag-pol* mRNA^+^/p24 protein^+^ infected cell per million CD4^+^ T cells (53, 54). The sensitivity of the assay is high, since a cell is reliably identified as *gag-pol* mRNA^+^ if it contains more than 20 copies of HIV-1 mRNA. Thus, this technique can distinguish infected cells from uninfected bystander cells with high specificity and sensitivity.

**FIG 8:**
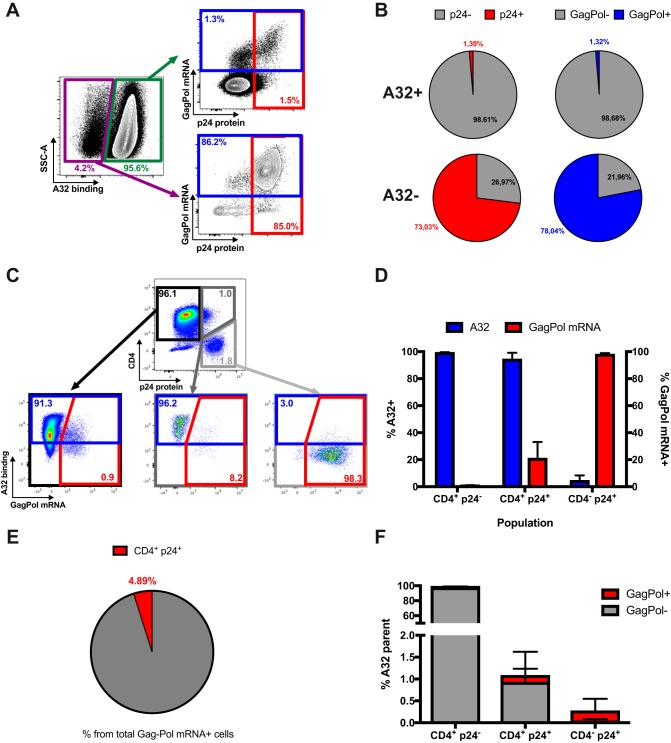
A32 preferentially binds to cells that are CD4^+^ p24^−^ *gag-pol* mRNA^−^. (A and B) Primary CD4^+^ T cells infected with NL3.4 ADA GFP WT virus were stained with A32, followed by appropriate secondary Abs. Cells were then stained for phenotypic markers (see Materials and Methods) prior to detection of HIV-1 p24 and *gag-pol* mRNA by RNA-flow FISH. (A) Example of flow cytometry gating strategy based on A32 binding. (B) Quantification of the percentage of cells positive for HIV-1 p24 or *gag-pol* mRNA based on A32 binding. (C) Example of flow cytometry gating based on p24 and CD4 expression. (D) Quantification of the percentage of cells positive for A32 binding and *gag-pol* mRNA based on CD4 and p24 levels. (E) Quantification of the percentage of p24^+^ CD4^+^ cells among the cells positive for *gag-pol* mRNA. (F) Quantification of the percentage of cells that are CD4^+^ p24^−^, CD4^+^ p24^+^, or CD4^−^ p24^+^ among the cells recognized by A32. Error bars indicate means ± standard errors of the means of at least 4 independent experiments.

For these experiments, primary CD4^+^ T cells were infected with the NL3.4 ADA GFP WT virus, and 48 h postinfection, the average percentage of infection was 8.0%. Infected cells were stained first with A32 before staining for phenotypic markers, such as CD4. Cells were then fixed and permeabilized to allow detection of the HIV-1 p24 antigen and *gag-pol* mRNA. We first tested whether CD4^+^ T cells recognized by A32 were positive for *gag-pol* mRNA (Fig. 8A and B) but found that less than 2% of these cells contained p24 protein or *gag-pol* mRNA. In contrast, the vast majority of A32-negative cells were positive for p24 protein (73.03%) or *gag-pol* mRNA (78.04%). This confirmed that the vast majority of CD4^+^ T cells recognized by A32 are uninfected bystander cells.

It remained possible, however, that the cells detected as p24^−^ *gag-pol* mRNA^−^ were in a very early stage of infection, before viral protein and mRNA could be detected. Indeed, previous studies have suggested that A32-like epitopes become transiently exposed during viral entry (55, 56). To investigate this possibility, uninfected bystander (GFP^−^) cells were sorted by flow cytometry to determine how many could become productively infected. After 5 additional days in cell culture, less than 3% of sorted GFP^−^ cells became infected (Fig. S7). Thus, most cells recognized by A32 are neither productively infected nor in a very early stage of infection.

10.1128/mBio.00358-18.8 FIG S7  The vast majority of uninfected bystander CD4^+^ T cells remain uninfected after 5 days in culture. Download FIG S7, PDF file, 0.7 MB. Copyright © 2018 Richard et al. 2018 Richard et al. This content is distributed under the terms of the Creative Commons Attribution 4.0 International license.

Since Env-CD4 interaction is critical for exposure of the A32 epitope (5–7), we next analyzed the RNA-flow FISH results based on p24 and CD4 expression. As shown in Fig. 8C and D, CD4^+^ p24^−^ cells were efficiently recognized by A32 (blue bars) but remained almost exclusively negative for *gag-pol* mRNA (red bars). Inversely, the CD4^−^ p24^+^ population was largely positive for *gag-pol* mRNA but was not recognized by A32. More recent studies suggested that the A32 epitope could be exposed on a fraction of p24^+^ cells because of residual CD4 expression (42, 57, 58). Therefore, we next quantified the recognition by A32 and infection of these p24^+^ CD4^+^ cells by RNA-flow FISH (dark gray box, Fig. 8C). Although this rare population was indeed recognized, only a fraction of A32-positive cells were productively infected (~20%). Similar results were obtained with primary CD4^+^ T cells infected with an X4-tropic virus (Fig. S8). Finally, we determined the proportion of *gag-pol* mRNA^+^ cells that were both p24^+^ and CD4^+^ and found less than 5% of such cells that could be recognized by A32 (Fig. 8E). We also performed the reverse analysis by first identifying A32^+^ CD4^+^ T cells and then determining how many of those cells were both p24 and CD4 positive. Since only ~1% of such cells were CD4^+^ p24^+^ (Fig. 8F), it seems clear that A32^+^ cells represent only a minuscule fraction of productively HIV-1-infected CD4 T cells.

10.1128/mBio.00358-18.9 FIG S8  Characterization of cells infected with the X4-tropic NL4.3 virus by the RNA-flow FISH method. Download FIG S8, PDF file, 0.7 MB. Copyright © 2018 Richard et al. 2018 Richard et al. This content is distributed under the terms of the Creative Commons Attribution 4.0 International license.

**FIG 9:**
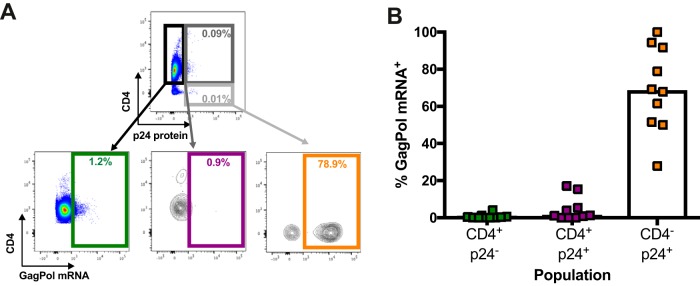
The CD4^+^ p24^+^ cell population represents a minimal fraction of the *gag-pol* mRNA^+^ cells in HIV-1-infected individuals. CD4^+^ T cells isolated from chronically HIV-infected, untreated individuals were rested overnight. Cells were then stained for phenotypic markers (see Materials and Methods) prior to detection of HIV-1 p24 and *gag-pol* mRNA by RNA-flow FISH. (A) Example of flow cytometry gating based on p24 and CD4 expression. (B) Quantification of the percentage of cells positive for *gag-pol* mRNA based on CD4 and p24 levels. Error bars indicate means ± standard errors of the means of data obtained with 10 HIV-1-infected individuals.

To determine whether *gag-pol* mRNA-containing CD4^+^ p24^+^ cells were present in the peripheral blood of HIV-1-infected individuals, we isolated CD4^+^ T cells from the blood of untreated chronically HIV-1-infected individuals, rested them overnight without stimulation, and then performed the RNA-flow FISH assay (Fig. 9A and B). Again, the CD4^+^ p24^+^ cell population represented only a minimal fraction of the *gag-pol* mRNA^+^ cells. Therefore, *in vivo* A32 is unlikely to recognize most HIV-1-infected cells.

## DISCUSSION

The conformation adopted by Env at the cell surface has considerable influence on Ab recognition and ADCC responses (2). In its unliganded form, Env from most primary virus samples adopts a “closed” trimeric conformation, preferentially recognized by bNAbs but not by CD4i Abs, which are abundant in plasma from most HIV-1-infected individuals (2, 4, 10, 15–18, 23, 59, 60, 86). One of the mechanisms that HIV-1 has developed to avoid exposing Env CD4i epitopes is the downregulation of CD4 cell surface expression. This is achieved in a two-step process. First, during the early phases of the HIV-1 replication cycle, Nef downregulates CD4 from the plasma membrane. Second, Vpu, expressed from a bicistronic mRNA also coding for Env, induces CD4 degradation through an endoplasmic reticulum (ER)-associated protein degradation (ERAD) mechanism in the ER (61). The action of Vpu liberates Env from CD4-dependent retention in the ER (62), allowing its trafficking to the plasma membrane in a “closed” conformation in which CD4i epitopes are occluded by oligomerization. These epitopes, however, are exposed in shed gp120 monomers that are released by the dissociation of the noncovalent gp120-gp41 interactions. Interestingly, *in vitro* experiments have shown that the binding of shed gp120 to uninfected bystander CD4^+^ T cells enables recognition of these cells by CD4i antibodies (16). Of note, this was seen using a variety of HIV-1 variants, including primary or transmitted founder viruses (Fig. 1 and S2) (16, 17, 58, 63), as well as simian-human immunodeficiency virus (SHIV) infectious molecular clones (16).

Here, we demonstrate that the uninfected bystander CD4^+^ T cell population, which is coated with shed gp120, represents a confounding factor when measuring ADCC responses *in vitro*. Using assays that are unable to differentiate infected from uninfected cell populations, we observed strong killing mediated by A32 and HIV^+^ sera (Fig. 3). This ADCC activity was not correlated with the inability of these antibodies to recognize infected cells (Fig. 1 and 4). Replacement of gp120-coated uninfected bystander CD4^+^ T cells with autologous mock-infected cells confirmed that most of the detected activities were directed against uninfected CD4^+^ T cells (Fig. 5 and 6). Using a sensitive RNA-flow FISH method, we next showed that A32 preferentially recognizes CD4^+^ cells that are negative for HIV-1 p24 and *gag-pol* mRNA (Fig. 8), while fewer than 2% of productively infected cells (p24^+^ *gag-pol* mRNA^+^) were recognized by this antibody. Although this population remains to be defined further, these cells likely represent virus-coated cells on which the A32 epitope has been transiently exposed as a result of the high density of Env-CD4 interactions, a possibility supported by the fact that they do not form a distinct population in FACS analyses but form a shoulder of the p24-negative population. The extent to which this cell population exists *in vivo*, and the ability of Fcγ receptor-bearing cells to gain access to CD4i epitopes, remains unknown. In contrast, the vast majority of productively infected cells were CD4^−^, both *in vitro* and in *ex vivo* samples from HIV-1-infected individuals. Consistent with poor recognition of infected cells by A32 and HIV^+^ sera, *in vitro* assays able to determine ADCC responses against infected cells failed to detect robust ADCC responses mediated by these ligands (Fig. 2). This was not due to a lack of sensitivity, since these assays readily detected ADCC responses mediated by the bNAbs PGT126 and 3BNC117 (Fig. 2). Thus, assays measuring responses on the total population missed the ADCC activity mediated by these bNAbs.

The results of our study highlight the difficulties in selecting an appropriate assay to measure ADCC. If ADCC is measured on the total population (granzyme B and NK cell activation), A32 and HIV^+^ sera appear to mediate a stronger ADCC response than PGT126 and 3BNC117. On the other hand, assays that can evaluate responses against infected cells show the opposite: PGT126 and 3BNC117 mediate significantly higher ADCC responses than A32 and HIV^+^ sera. It seems clear that ligand recognition of gp120-coated uninfected bystander CD4^+^ T cells is, at least in part, responsible for these differences. Indeed, removal of these cells significantly reduced the ADCC activity detected for A32 and HIV^+^ sera. Therefore, the differential recognition of the uninfected bystander cell population by any given ligand has a significant impact on *in vitro* ADCC measurements. It is well established that HIV-1 accessory proteins Nef and Vpu protect HIV-1-infected cells from ADCC responses (5, 7, 10, 15, 19–21, 64, 65). Assays measuring the elimination of infected cells were able to confirm these observations (Fig. 2, S3, and S4), while those that measure the total population (granzyme B and NK cell activation) failed to do so (Fig. 3). Thus, the presence of gp120-coated uninfected bystander CD4^+^ T cells confounds *in vitro* ADCC measurements.

Previous reports demonstrated that the majority of ADCC activity present in HIV^+^ sera is mediated by anti-cluster A antibodies (9–11, 14). These antibodies preferentially target Env in its CD4-bound conformation (5, 8). Intriguingly, we observed variable ADCC activity among the different HIV^+^ sera tested (Fig. 2, 3, and 6). It is possible that differences in their concentration in sera account for some of this variability. However, we cannot rule out that the presence of additional ADCC-mediating Abs that do not require the CD4-bound conformation of Env to recognize infected cells, or that target the gp41, might also contribute to this variable ADCC activity.

While passive administration of ADCC-mediating nnAbs, including A32, has failed to protect macaques against simian immunodeficiency virus (SIV) or SHIV challenges (66–70), several studies have identified ADCC responses measured against total cell population or gp120-coated target cells as correlates of protection in these same animal models (45, 71–74). Moreover, CD4i vaccines have been reported to protect macaques from viral challenge (45, 72). Since Env conformation greatly influences ADCC responses (8), it is possible that the conformation of Env in the challenge viruses impacted the reported protection efficacy. It is conceivable that the Env of these challenge stocks sampled a slightly more “open” conformation, readily recognized by CD4i Abs but not present in primary viruses (8). For example, nonneutralizing CD4i Abs with ADCC activity, in the presence of low levels in plasma of IgA Env-specific Abs, inversely correlated with HIV-1 acquisition in the RV144 trial (75). A recent study suggested that the presence of a naturally occurring histidine at position 375 (H375) in the Phe 43 cavity of the predominant strain (CRF01_AE) replicating in Thailand might have contributed to the efficacy of the trial by spontaneously exposing epitopes recognized by ADCC-mediating antibodies elicited by the RV144 vaccine regimen (76). Our results warrant further studies to assess the conformation of Envs of current SHIVs used in vaccine efficacy studies.

Since Env conformation and the nature of target cells greatly influence ADCC results, our study highlights the need for careful assay selection. Assays measuring ADCC responses on the total cell population (Fig. 3 to 6) or using target cells coated with recombinant gp120 (Fig. 7) or infected with viruses defective for Nef and Vpu expression (Fig. 2) favor the detection of ADCC responses mediated by CD4i Abs over those induced by bNAbs. Assays measuring ADCC responses on the infected-cell population are better suited to evaluate responses mediated by Abs recognizing the CD4-binding site or trimeric Env. Thus, these parameters must be carefully considered before selecting assays for characterizing HIV-1-specific ADCC responses when evaluating responses mediated by monoclonal Abs, mechanisms of immune evasion, or correlates of vaccine protection.

## MATERIALS AND METHODS

### Ethics statement.

Written informed consent was obtained from all study participants (the Montreal Primary HIV Infection Cohort [77, 78] and the Canadian Cohort of HIV Infected Slow Progressors [79–81]), and research adhered to the ethical guidelines of the Centre de Recherche du CHUM (CRCHUM) and was reviewed and approved by the CRCHUM institutional review board (ethics committee approval number CE 16.164-CA). Research adhered to the standards indicated by the Declaration of Helsinki. All participants were adult and provided informed written consent prior to enrollment in accordance with institutional review board approval.

### Cell lines and isolation of primary cells.

HEK293T human embryonic kidney cells (obtained from ATCC) and CEM.NKr-CCR5-sLTR-Luc cells were grown as previously described (7, 15). Primary human PBMCs, NK cells, and CD4^+^ T cells were isolated, activated, and cultured as previously described (7, 15) and detailed in the supplemental material.

### Viral production and infections.

To achieve the same level of infection among the different IMCs (infectious molecular clones) tested, vesicular stomatitis virus G (VSVG)-pseudotyped HIV-1 viruses were produced and titrated as previously described (5). Viruses were then used to infect activated primary CD4 T cells from healthy HIV-1-negative donors or CEM.NKr-CCR5-sLTR-Luc cells by spin infection at 800 × *g* for 1 h in 96-well plates at 25°C.

### Antibodies and sera.

A detailed list of the Abs used for cell surface staining, ADCC measurement, and RNA flow analysis is presented in the supplemental material. Sera from HIV-infected and uninfected donors were collected, heat inactivated, and conserved as previously described (7, 15). A random number generator (QuickCalcs; GraphPad, San Diego, CA) was used to randomly select a number of sera for each experiment.

### Plasmids and site-directed mutagenesis.

pNL43-ADA(Env)-GFP.IRES.Nef proviral vectors containing intact or defective *nef* and *vpu* genes, as well as the VSVG-encoding plasmid (pSVCMV-IN-VSV-G), were previously described (5). The plasmid encoding the HIV-1 transmitted founder (TF) IMC CH77 containing intact or defective *nef* and *vpu* genes was previously described (10, 15, 82–85).

### Flow cytometry analysis of cell surface staining.

Cell surface staining was performed as previously described (5, 15). Binding of HIV-1-infected cells by sera (1:1,000 dilution), anti-Env MAbs (A32, PGT126, or 3BNC117) (5 μg/ml), or anti-CD4 MAbs (1 μg/ml) was performed at 48 h postinfection. Cells infected with HIV-1 primary isolates were stained intracellularly for HIV-1 p24, using the Cytofix/Cytoperm fixation/permeabilization kit (BD Biosciences, Mississauga, ON, Canada) and the fluorescent anti-p24 MAb (phycoerythrin [PE]-conjugated anti-p24, clone KC57; Beckman Coulter/Immunotech). The percentage of infected cells (p24^+^ or GFP^+^ cells) was determined by gating the living cell population on the basis of the AquaVivid viability dye staining. Samples were analyzed on an LSR II cytometer (BD Biosciences), and data analysis was performed using FlowJo vX.0.7 (Tree Star, Ashland, OR, USA).

### Replacement of uninfected bystander cells by autologous mock cells.

Uninfected bystander cells (GFP^−^ CD4^high^ T cells) were removed from the target cell population using the Dynabeads CD4-positive selection kit (Invitrogen) at a ratio of 25 μl of beads per million cells. Enrichment of infected primary GFP^+^ CD4^low^ T cells was assessed by cell surface staining with the anti-CD4 OKT4 Ab (Fig. 5A). Uninfected bystander cells were then replaced by the same number of autologous mock cells prior to staining with A32 or performing ADCC measurements.

### ADCC measurements.

ADCC responses were measured at 48 h postinfection, as described in detail in the supplemental material. For the FACS-based, granzyme B, and NK cell activation assays, MAbs were used at 5 μg/ml and human sera were used at a 1:1,000 dilution. For the luciferase assay, MAbs were used at 0.0024, 0.0098, 0.0390, 0.1563, 0.6250, 2.5, 10, or 40 μg/ml and human sera were used at a dilution of 1:100, 1:400, 1:1,600, 1:6,400, 1:25,600, 1:102,400, 1:409,600, or 1:1,638,400. For the RFADCC assay, MAbs were used at 0.008, 0.04, 0.2, 1, and 5 μg/ml and human sera were used at a dilution of 1:100, 1:400, 1:1,600, 1:6,400, or 1:25,600.

### RNA-flow analysis.

Samples were processed using the HIV RNA/Gag RNA flow assay as previously described (53, 54). Briefly, for *in vitro* studies, primary CD4^+^ T cells infected for 48 h were collected and indirectly surface stained for HIV Env using A32 (as described above) before further staining for phenotypic markers. For *ex vivo* studies, CD4^+^ T cells were isolated from chronically HIV-infected, untreated individuals and rested overnight in the presence of antiretrovirals (zidovudine [AZT] plus T20) in order to block new cycles of infection. In all experiments, cells were labeled with a viability dye (eFluor 506 fixable viability dye; ThermoFisher Scientific) and surface stained for phenotypic markers (CD3, CD4, and exclusion [CD8, CD14, and CD19]), before fixation, permeabilization, and intracellular staining for HIV p24. HIV *gag-pol* mRNA was labeled using the ThermoFisher PrimeFlow kit using probes designed against JR-CSF (53, 54). Samples were acquired on a BD LSR II cytometer (BD Biosciences), and data analysis was performed using FlowJo vX.0.7 (Tree Star).

### Statistical analyses.

Statistics were analyzed using GraphPad Prism version 6.01 (GraphPad, San Diego, CA). Every data set was tested for statistical normality, and this information was used to apply the appropriate (parametric or nonparametric) statistical test. *P* values of <0.05 were considered significant.

10.1128/mBio.00358-18.1 TEXT S1  Supplemental methods. Download TEXT S1, PDF file, 0.1 MB. Copyright © 2018 Richard et al. 2018 Richard et al. This content is distributed under the terms of the Creative Commons Attribution 4.0 International license.
