# Supplementary figures and images for: Uninfected Bystander Cells Impact the Measurement of HIV-Specific Antibody-Dependent Cellular Cytotoxicity Responses
Source: mBio. 2018 Mar 20;9(2):e00358-18. doi: 10.1128/mBio.00358-18 (PMC5874913; doi:10.1128/mBio.00358-18)

**A**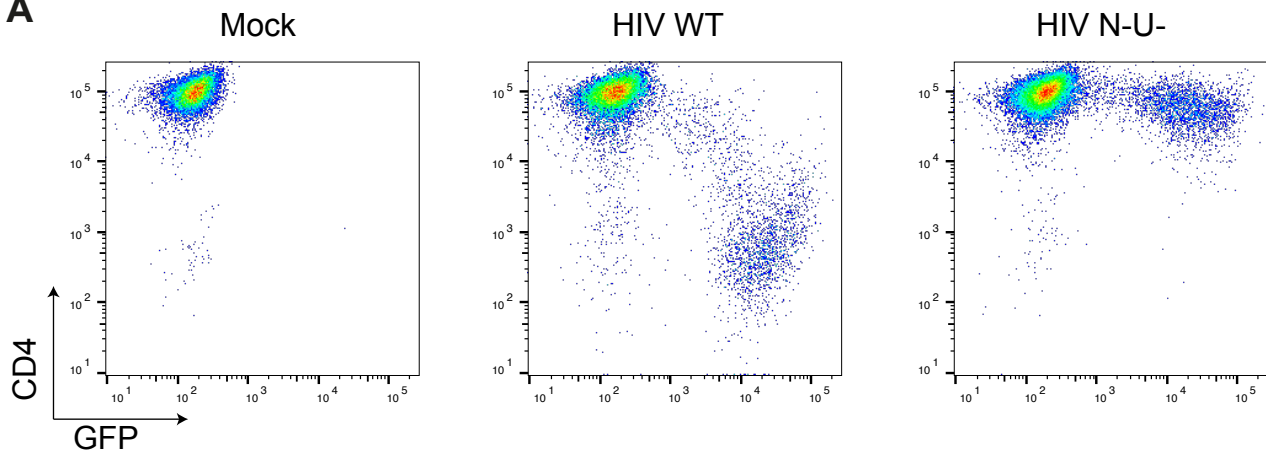**B**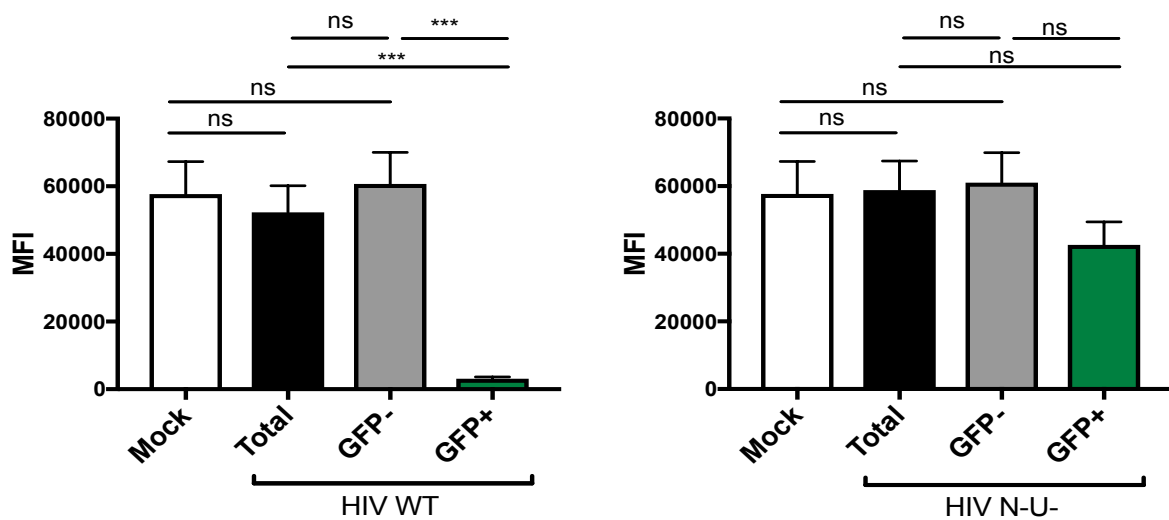

Supplement: FIG S1 [file mbo002183786sf1.pdf]

**A**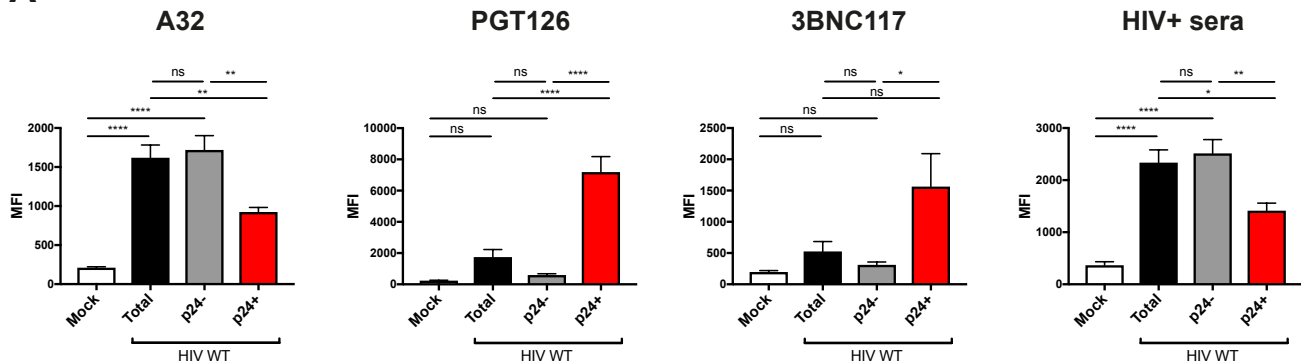**B**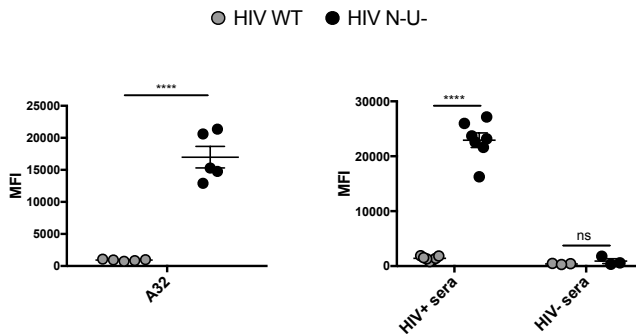

Supplement: FIG S2 [file mbo002183786sf2.pdf]

**A**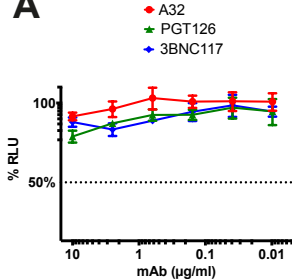**B**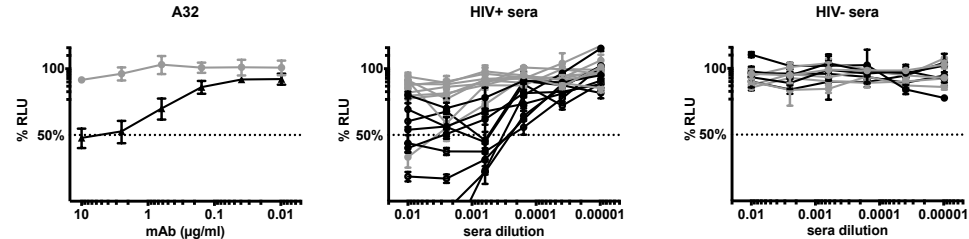

Supplement: FIG S3 [file mbo002183786sf3.pdf]

**A**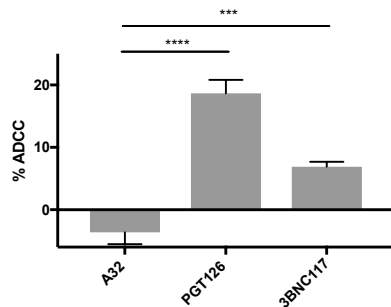**B**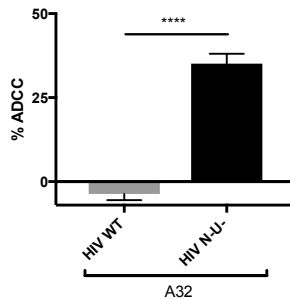**C**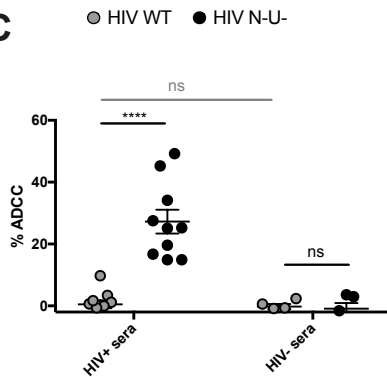**D**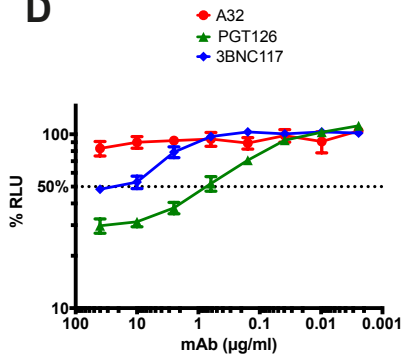**E**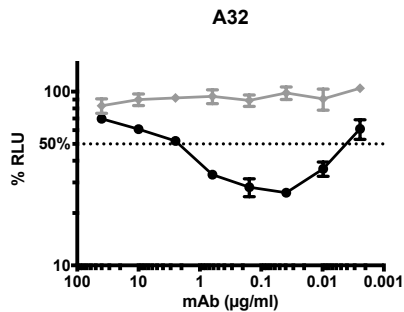**F**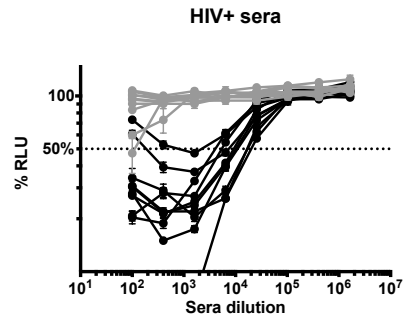

Supplement: FIG S4 [file mbo002183786sf4.pdf]

**A****HIV WT****HIV N-U-**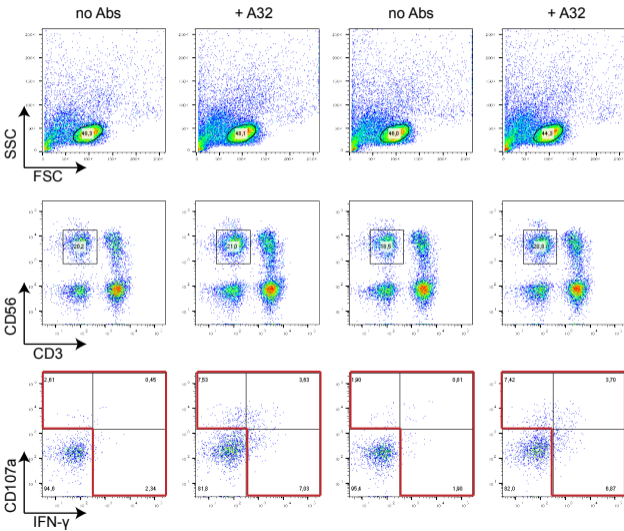**B****HIV WT****HIV N-U-**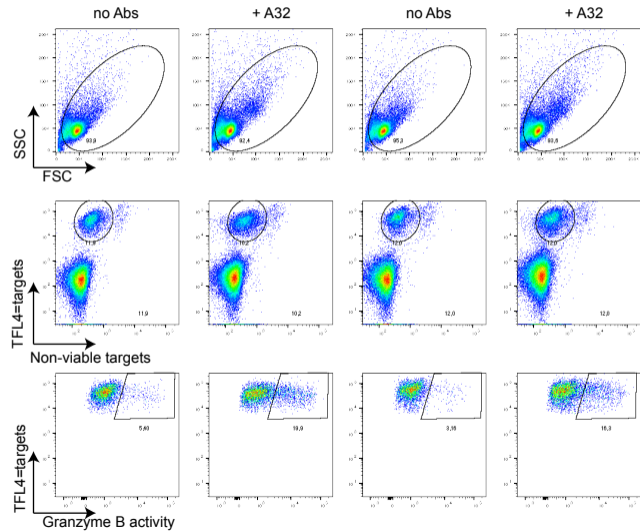

Supplement: FIG S5 [file mbo002183786sf5.pdf]

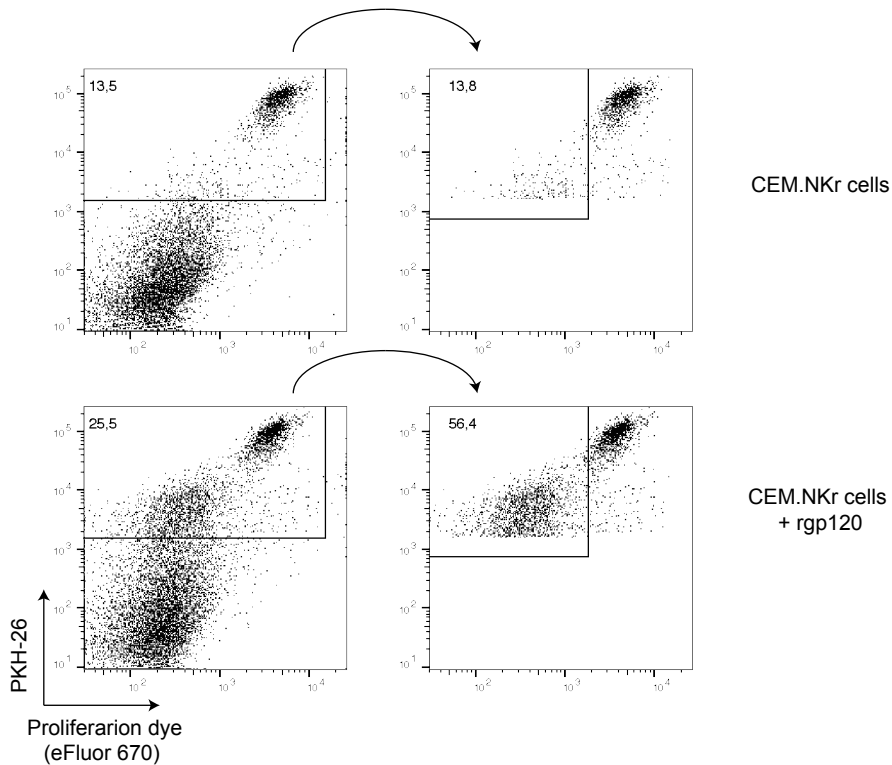

Supplement: FIG S6 [file mbo002183786sf6.pdf]

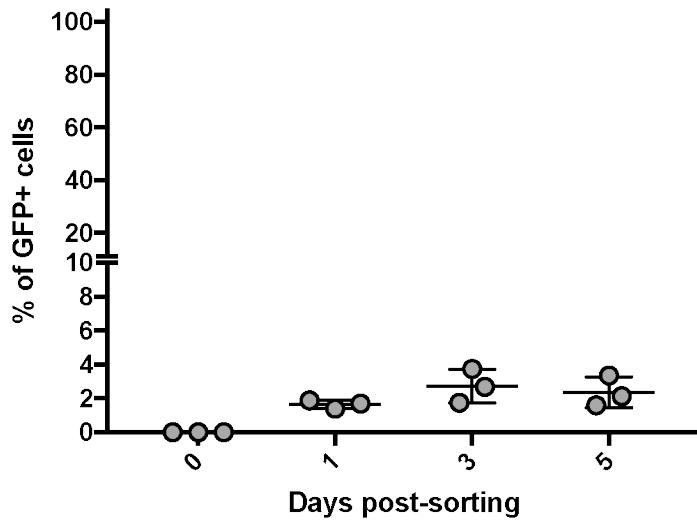

Supplement: FIG S7 [file mbo002183786sf7.pdf]

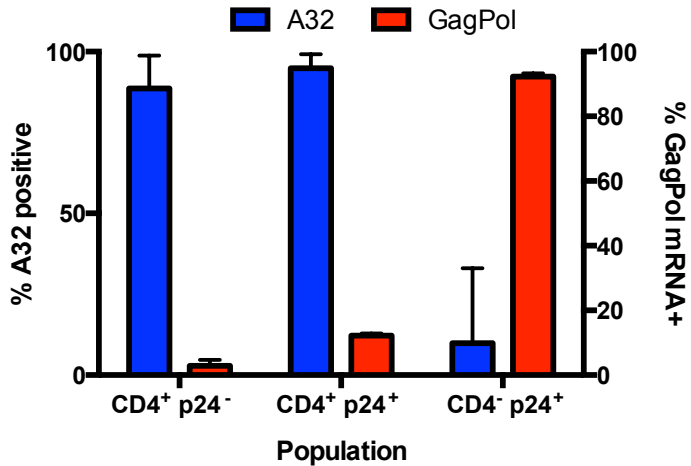

Supplement: FIG S8 [file mbo002183786sf8.pdf]
