# Supplementary material for: Uninfected Bystander Cells Impact the Measurement of HIV-Specific Antibody-Dependent Cellular Cytotoxicity Responses
Source: mBio. 2018 Mar 20;9(2):e00358-18. doi: 10.1128/mBio.00358-18 (PMC5874913; doi:10.1128/mBio.00358-18)
Supplement: TEXT S1 [file mbo002183786s1.pdf]

SUPPLEMENTAL METHODS

Primary cells

PBMC were obtained by leukapheresis. NK cells and CD4+ T lymphocytes were purified from resting PBMCs by negative selection using immunomagnetic beads per the manufacturer's instructions (StemCell Technologies, Vancouver, BC). NK cells were cultured overnight in RPMI 1640 complete medium supplemented with 20% fetal bovine serum and 100 μg/mL penicillin-streptomycin before use. CD4+ T lymphocytes were activated with phytohemagglutinin-L (10 μg/ mL) for 48 hours and then maintained in RPMI 1640 complete medium supplemented with rIL-2 (100 U/mL).

Antibodies

The following Abs were used as first Ab for cell surface staining: 1 μg/mL mouse anti-CD4 mAb OKT4 (14-0048-82; eBiosciences), 5 μg/mL human anti-HIV-1 Env mAbs A32, PGT126 (kindly provided by the International AIDS Vaccine Initiative), 3BNC117 (kindly provided by M.C. Nussenzweig, The Rockefeller University, New York, NY), 1 μg/mL of either Alexa Fluor 647-conjugated goat anti-mouse or goat anti-human Abs (Thermo Fisher Scientific) were used as secondary Abs. The following Abs were used for the NK cell activation assay: APC-conjugated anti-CD107a (BD Biosciences, H4A3), BV421-conjugated anti-CD3 (Biolegend, UCHT1), PE-conjugated anti-CD56 (BD Biosciences, NCAM16.2) and PE-Cy7-conjugated anti-IFNγ (BD Biosciences, B27). The following Abs were used for RNA-flow analysis: BUV395-conjugated anti-CD3 (BD Biosciences, UCHT1), PE-Cy7-conjugated anti-CD4 (BD Biosciences, RPA-T4) BV510-

conjugated anti-CD8 (Biolegend, SK1), BV510-conjugated anti-CD14 (Biolegend, M5E2) BV510-conjugated anti-CD19 (Biolegend, H1B19) and PE-conjugated anti-p24 (Beckman Coulter/Immunotech, KC57), while AquaVivid (Thermo Fisher Scientific) was used as viability dye.

ADCC measurements

FACS-based assay

Measurement of ADCC using the FACS-based assay was performed at 48h post-infection as previously described (1-3). Briefly, infected primary CD4+ T cells were stained with viability (AquaVivid; Thermo Fisher Scientific) and cellular (cell proliferation dye eFluor670; eBioscience) markers and used as target cells. Autologous PBMC effectors cells, stained with another cellular marker (cell proliferation dye eFluor450; eBioscience), were added at an effector: target ratio of 10:1 in 96-well V-bottom plates (Corning, Corning, NY). A 1:1,000 final dilution of sera or 5 μg/ml of ADCC-mediating mAbs were added to appropriate wells and cells were incubated for 15 min at room temperature. The plates were subsequently centrifuged for 1 min at 300 g, and incubated at 37°C, 5% CO_2 for 5 to 6 h before being fixed in a 2% PBS-formaldehyde solution containing $5x10^4$ flow cytometry particles/ml (AccuCount blank particles; 5.3 m; Spherotech). Samples were analyzed on an LSRII cytometer (BD Biosciences) and acquisition was set to acquire 1,000 flow cytometry particles, which allows the calculation of relative cell counts (2). Data analysis was performed using FlowJo vX.0.7 (Tree Star). The percentage of ADCC was calculated with the following formula: (relative count of GFP+ cells in targets plus effectors) - (relative count of GFP+

cells in targets plus effectors plus Abs or sera) / (relative count of GFP+ cells in targets) by gating infected live target cells. For cells infected with HIV-1 primary isolates, infected cells were identified by intracellular staining for HIV-1 p24. In that context, the percentage of ADCC was calculated with the following formula: (% of p24+ cells in Targets plus Effectors) − (% of p24+ cells in Targets plus Effectors plus sera) / (% of p24+ cells in Targets) by gating on infected lived target cells.

Granzyme B assay

Measurement of ADCC response using the Granzyme B assay was performed 48h post-infection according to the manufacturer's instructions (OncoImmunin, Gaithersburg, MD) and as previously described (4, 5). Briefly, infected primary CD4+ T cells were stained with a fluorescent target cell marker (TFL4; OncoImmunin, Gaithersburg, MD) and a viability marker (NFL1; OncoImmunin or AquaVivid; Thermo Fisher Scientific). Rested autologous purified NK cells were used as effector cells. Target and effector cells were counted and adjusted to reach a final effector: target ratio of 10:1. Twenty-five μl of each effector and target cell suspension and 75 μl of Granzyme B substrate (OncoImmunin) was dispensed into each well of a 96-well V-bottom plate (Corning). A 1:1,000 final dilution of sera or 5 μg/ml or ADCC-mediating Abs were added to appropriate wells and cells were incubated for 15 min at room temperature. The plates were subsequently centrifuged for 1 min at 300 g, and incubated for 1 h at 37°C and 5% CO_2. After two washes, cells were acquired on an LSRII cytometer (BD Biosciences) and data analysis was performed using FlowJo vX.0.7 (Tree Star). The percentage of Ab or sera-induced Granzyme B activity was calculated with the following formula:

(Percentage of Granzyme B+ target cells in targets plus effectors plus Abs or sera) - (Percentage of Granzyme B+ target cells in targets plus effectors) per the gating strategy presented in Fig S5.

NK cell activation assay

Measurement of ADCC response using the NK cell activation assay was performed using a modified version of a previously described protocol (6). Briefly, primary CD4+ T cells infected for 48h were co-cultured with autologous PBMC at an effector: target ratio of 5:1 in the presence of a 1:1,000 final dilution of sera or 5 μg/ml or ADCC-mediating Abs, anti-CD107a, Brefeldin A (Sigma) (5μg/ml) and Monensin (BD Biosciences) (6μg/ml) for 5h at 37°C. Control conditions included incubation of PBMC alone or incubation of PBMC with mock cells in the presence of sera or ADCC-mediating Abs. After incubation cells were surface stained with anti-CD3 and anti-CD56 Abs. Next, cells were fixed and permeabilized using the Cytofix/Cytoperm Fixation/ Permeabilization Kit (BD Biosciences) and stained with anti-IFNγ Ab. Samples were analyzed on an LSRII cytometer (BD Biosciences) and data analysis was performed using FlowJo vX.0.7 (Tree Star). The percentage of Ab or sera-induced NK cell activation was calculated with the following formula: (Percentage of NK cells (CD3-CD56+) positive for CD107a and/or IFNγ in targets plus effectors plus Abs or sera) - (Percentage of NK cells (CD3-CD56+) positive for CD107a and/or IFNγ in targets plus effectors) according to the gating strategy presented in Fig S5.

Luciferase assay

Measurement of ADCC responses using the Luciferase assay was performed as previously described (7). Briefly, CEM.NKR-CCR5-sLTR-Luc target cells, which express luciferase (Luc) under the control of a Tat-inducible promoter, were infected and used as target cells at 48h post-infection. Primary human PBMCs or NK cell line expressing human CD16 were used as effector cells. Target and effector cells were co-cultured for 6-8h, in triplicate, at an effector: target ratio of 10:1 in the presence of different concentrations of Abs (0.0024, 0.0098, 0.0390, 0.1563, 0.6250, 2.5, 10 or 40 μg/ml) or different dilution of human sera (1:100, 1:400, 1:1600, 1:6400, 1:25600, 1:102400, 1:409600 or 1:1638400). The dose-dependent loss of Luc activity was measured as an indication of sera or Ab-mediated killing of productively-infected cells. Infected target cells incubated with effector cells in the absence of sera or Ab were used to measure maximal Luc activity, and uninfected target cells cultured with effector cells were used to determine background Luc activity. Area under the curve (AUC) values for ADCC were calculated as previously described (7). For experiments involving CH77 wt and N-U- viruses, the *vif* gene was deleted by site-directed mutagenesis. The nucleotides 62 and 63 in *vif* were deleted, causing a frame-shift that resulted in an early stop codon (W21*) while preserving the *pol* open reading frame.

RFADCC assay

CEM.NKR-CCR5-sLTR-Luc target cells were coated or not with 1 μg/ml of recombinant gp120 protein derived from the HIV-1 YU2 strain for 30 minutes at 37°C +5% CO_2. Cells were then double stained with PKH26 red fluorescent cell linker (Sigma-Aldrich, USA) and the cellular proliferation marker eFluor670 (eBioscience) as

previously described (2, 8, 9) Cells were then dispensed together with different concentration of Abs (0.008, 0.04, 0.2, 1 and 5 μg/ml) or different dilutions of sera (1:100, 1:400, 1:1600, 1:6400 and 1:25600). After 15 min at room temperature, effector cells (Rested primary PBMCs) were added at an effector: target ratio of 50:1. Plates were centrifuged for 1 min at 300 g and incubated for 4 h at 37°C. Cells were washed, fixed and acquired on an LSRII cytometer (BD Biosciences). Data analysis was performed using FlowJo vX.0.7 (Tree Star). The percentage of cytotoxicity was calculated with the following formula: (Percentage of cells that remained PKH26+ but that had lost the proliferation dye (eFluor670-) in target cells coated with recombinant gp120 plus effectors plus Abs or sera) - (Percentage of cells that remained PKH26+ but that had lost the proliferation dye (eFluor670-) in target cells plus effectors plus Abs or sera) according to the gating strategy presented in Fig S6.

References

1. Veillette M, Desormeaux A, Medjahed H, Gharsallah NE, Coutu M, Baalwa J, Guan Y, Lewis G, Ferrari G, Hahn BH, Haynes BF, Robinson JE, Kaufmann DE, Bonsignori M, Sodroski J, Finzi A. 2014. Interaction with cellular CD4 exposes HIV-1 envelope epitopes targeted by antibody-dependent cell-mediated cytotoxicity. J Virol 88:2633-44.
2. Richard J, Veillette M, Batraville LA, Coutu M, Chapleau JP, Bonsignori M, Bernard N, Tremblay C, Roger M, Kaufmann DE, Finzi A. 2014. Flow cytometry-based assay to study HIV-1 gp120 specific antibody-dependent cellular cytotoxicity responses. J Virol Methods 208:107-14.
3. Richard J, Veillette M, Brassard N, Iyer SS, Roger M, Martin L, Pazgier M, Schon A, Freire E, Routy JP, Smith AB, 3rd, Park J, Jones DM, Courter JR, Melillo BN, Kaufmann DE, Hahn BH, Permar SR, Haynes BF, Madani N, Sodroski JG, Finzi A. 2015. CD4 mimetics sensitize HIV-1-infected cells to ADCC. Proc Natl Acad Sci U S A 112:E2687-94.
4. Pollara J, Hart L, Brewer F, Pickeral J, Packard BZ, Hoxie JA, Komoriya A, Ochsenbauer C, Kappes JC, Roederer M, Huang Y, Weinhold KJ, Tomaras GD, Haynes BF, Montefiori DC, Ferrari G. 2011. High-throughput quantitative analysis of HIV-1 and SIV-specific ADCC-mediating antibody responses. Cytometry A 79:603-12.
5. Konstantinus IN, Gamieldien H, Mkhize NN, Kriek JM, Passmore JA. 2016. Comparing high-throughput methods to measure NK cell-mediated antibody

dependent cellular cytotoxicity during HIV-infection. J Immunol Methods 434:46-52.

6. Gooneratne SL, Richard J, Lee WS, Finzi A, Kent SJ, Parsons MS. 2015. Slaying the Trojan horse: natural killer cells exhibit robust anti-HIV-1 antibody-dependent activation and cytolysis against allogeneic T cells. J Virol 89:97-109.

7. Alpert MD, Heyer LN, Williams DE, Harvey JD, Greenough T, Allhorn M, Evans DT. 2012. A novel assay for antibody-dependent cell-mediated cytotoxicity against HIV-1- or SIV-infected cells reveals incomplete overlap with antibodies measured by neutralization and binding assays. J Virol 86:12039-52.

8. Gomez-Roman VR, Florese RH, Patterson LJ, Peng B, Venzon D, Aldrich K, Robert-Guroff M. 2006. A simplified method for the rapid fluorometric assessment of antibody-dependent cell-mediated cytotoxicity. J Immunol Methods 308:53-67.

9. Ruiz MJ, Ghiglione Y, Falivene J, Laufer N, Holgado MP, Socias ME, Cahn P, Sued O, Giavedoni L, Salomon H, Gherardi MM, Rodriguez AM, Turk G. 2016. Env-Specific IgA from Viremic HIV-Infected Subjects Compromises Antibody-Dependent Cellular Cytotoxicity. J Virol 90:670-81.

SUPPLEMENTAL FIGURE LEGEND

Fig S1. Level of cell-surface CD4 on infected and uninfected cells. Cell-surface staining of primary CD4+ T cells mock-infected (Mock) or infected with the NL4.3 ADA GFP virus, either wild-type (HIV WT) or defective for Nef and Vpu expression (HIV N-U-) with the anti-CD4 OKT4 Ab (1μg/ml) (A) Dot plots depicting representative staining. (B) Mean Fluorescence Intensity (MFI) obtained for at least 5 different experiments. Error bars indicate means ± standard errors of the means. Statistical significance was tested using Ordinary one-way ANOVA. (*** $p<0.001$, ns: non-significant).

Fig S2. Recognition of primary CD4+ T cells infected with the transmitted/founder virus CH77. Primary CD4+ T cells were mock-infected or infected with the transmitted/founder virus CH77, either wild-type (HIV WT) or defective for Nef and Vpu expression (HIV N-U-). Forty-eight hours post-infection, cells were stained with A32, PGT126, 3BNC117 (5 μg/ml) or sera from 10 HIV-1-infected individuals (HIV+ sera) or 5 uninfected individuals (HIV- sera) (1:1000 dilution), followed with appropriate secondary Abs. Shown in (A) are the mean fluorescence intensities (MFI) obtained for at least 5 independent stains with the different Abs, with 10 HIV+ or 5 HIV- sera on cells infected with WT virus. Graphs shown in (B) represent the MFI obtained for 5 independent stains with A32, with 10 HIV+ sera or 5 HIV- sera on cells infected with WT and N-U- virus. Error bars indicate means ± standard errors of the means. Statistical

significance was tested using (A) Ordinary one-way ANOVA, (B) unpaired t test or Mann-Whitney test (* p<0.05, (** p<0.01, **** p<0.0001, ns: non-significant).

Fig S3. ADCC responses detected by the Luciferase assays. Representation of the ADCC responses detected by the Luc assay (presented in Fig 2) as the loss of luciferase signal (RLU). (A) ADCC responses mediated by A32, PGT126 and 3BNC117 (0.0024, 0.0098, 0.0390, 0.1563, 0.6250, 2.5 and 10 μg/ml) against cells infected with NL4.3 ADA GFP WT. (B) ADCC responses mediated by A32 (0.0024, 0.0098, 0.0390, 0.1563, 0.6250, 2.5 and 10 μg/ml), HIV+ or HIV- sera (1:100, 1:400, 1:1600, 1:6400, 1:25600 and 1:102400) against cells infected with NL4.3 ADA GFP WT (HIV WT) or defective for Nef and Vpu expression (HIV N-U-). Error bars indicate means ± standard errors of the means.

Fig S4. ADCC responses detected with the FACS-based and Luciferase assays against cells infected with the transmitted/founder virus CH77. (A-C) Primary CD4+ T cells or (D-F) CEM.NKR-CCR5-sLTR-Luc cells infected with the transmitted/founder virus CH77, either wild-type (HIV WT) (depicted in gray) or defective for Nef and Vpu expression (HIV N-U-) (depicted in black) were used as target cells with the (A-C) FACS-based infected cell elimination assay or (D-F) the Luciferase assays. Shown in (A,D) are ADCC responses mediated by A32, PGT126 and 3BNC117 against cells infected with WT virus, and in (B,C,E,F) ADCC responses mediated by (B,E) A32 or (C,F) HIV+ and HIV- sera against cells infected with WT or N-U- virus. (A-C) All graphs shown represent ADCC responses obtained from at least 5 independent

experiments. (D-F) ADCC responses presented as loss of luciferase signal (RLU) in triplicate. For the FACS-based assay, mAbs were used at 5μg/ml and human sera at a 1:1000 dilution. For the Luciferase assay, mAbs were used at 0.0024, 0.0098, 0.0390, 0.1563, 0.6250, 2.5, 10 and 40 μg/ml and human sera at a dilution of 1:100, 1:400, 1:1600, 1:6400, 1:25600, 1:102400, 1:409600 and 1:1638400. Error bars indicate means ± standard errors of the means. Statistical significance was tested using unpaired t test (*** $p<0.001$, **** $p<0.0001$, ns: non-significant).

Fig S5. Gating strategy used for the NK cell activation and Granzyme B assays. Gating strategy and dot plots depicting a representative detection of (A) NK cell activation and (B) granzyme B activity in the context of cells infected with NL4.3 ADA GFP virus, either WT (HIV WT) or defective for Nef and Vpu expression (HIV N-U-) in the presence or absence of A32.

Fig S6. Gating strategy used for the RFADCC assays. Gating strategy and dot plots depicting representative detection of responses mediated by A32 against target cells coated or not with recombinant gp120 using the RFADCC assay.

Fig S7. The vast majority of uninfected bystander CD4+ T cells remain uninfected after 5 days in culture. Primary CD4+ T cells were infected with the NL4.3 ADA GFP WT. Forty-eight hours post-infection, the GFP- cell population was sorted by flow cytometry and kept in culture for 5 days. The vast majority of cells (>96%) remained

negative for GFP after 5 days of culture. Error bars indicate means ± standard errors of the means for 3 independent experiments.

Fig S8. Characterization of cells infected with the X4-tropic NL4.3 virus by RNA-Flow FISH method. Quantification of the percentage of cells positive for A32 binding and *Gag-Pol* mRNA based on CD4 and p24 levels among primary CD4+ T cells infected with the X4-tropic NL4.3 WT virus. Error bars indicate means ± standard errors of the means of at least 4 independent experiments.
